# The role of arousal in the spontaneous regulation of emotions in healthy aging: a fMRI investigation

**DOI:** 10.3389/fpsyg.2014.00681

**Published:** 2014-07-28

**Authors:** Sanda Dolcos, Yuta Katsumi, Roger A. Dixon

**Affiliations:** ^1^Department of Psychology, University of Illinois at Urbana-ChampaignChampaign, IL, USA; ^2^Department of Psychology, University of AlbertaEdmonton, AB, Canada

**Keywords:** emotion control, spontaneous emotion regulation, brain imaging

## Abstract

Despite ample support for enhanced affective well-being and emotional stability in healthy aging, the role of potentially important dimensions, such as the emotional arousal, has not been systematically investigated in neuroimaging studies. In addition, the few behavioral studies that examined effects of arousal have produced inconsistent findings. The present study manipulated the arousal of pictorial stimuli to test the hypothesis that preserved emotional functioning in aging is modulated by the level of arousal, and to identify the associated neural correlates. Young and older healthy participants were presented with negative and neutral pictures, which they rated for emotional content, while fMRI data were recorded. There were three main novel findings regarding the neural mechanisms underlying the processing of negative pictures with different levels of arousal in young and older adults. First, the common engagement of the right amygdala in young and older adults was driven by high arousing negative stimuli. Second, complementing an age-related reduction in the subjective ratings for low arousing negative pictures, there were opposing patterns of activity in the rostral/ventral anterior cingulate cortex (ACC) and the amygdala, which showed increased vs. decreased responses, respectively, to low arousing negative pictures. Third, increased spontaneous activity in the ventral ACC/ventromedial prefrontal cortex (vmPFC) in older adults was linked to reduced ratings for low arousing negative pictures. Overall, these findings advance our understanding of the neural correlates underlying processing of negative emotions with different levels of arousal in the context of enhanced emotional functioning in healthy aging. Notably, the results support the idea that older adults have emotion regulation networks chronically activated, in the absence of explicit induction of the goal to regulate emotions, and that this effect is specific to low arousing negative emotions.

## Introduction

Aging is associated with well-known co-morbidities and losses, but also with relatively high levels of emotional well-being. The idea of relatively well-preserved emotional processing in aging is supported by evidence showing that older adults tend to (a) pay attention to and remember more positive information (Charles et al., 2003; Mather and Carstensen, 2003; Isaacowitz et al., 2006) and (b) show reduced processing of negative information compared to young adults (Wood and Kisley, 2006; Gruhn et al., 2007).

Prominent models of emotion identify valence and arousal as fundamental components of emotion (see Bradley and Lang, 2000, for a review). Valence refers to the direction of an emotional response (positive or negative), whereas arousal refers to the magnitude of the response (exciting, agitating, or calming, subduing; Russell, 1980). Empirical evidence shows that these two emotional dimensions are not independent of each other (Ito et al., 1998; Lang et al., 1998; Libkuman et al., 2007; but see Ribeiro et al., 2007). Rather, they form a V (“boomerang”)-shaped function, with the unpleasant pictures tending to be more highly arousing than pleasant stimuli, and both pleasant and unpleasant pictures being more arousing than neutral stimuli. Considerable evidence supports the “boomerang” shape in averaged data on arousal and valence ratings of people's reactions to affective visual stimuli, such as the International Affective Picture System (IAPS) (e.g., Lang et al., 1992, 1998; Lang, 1995; Bradley and Lang, 2007). Despite substantial support for the preserved emotional function in aging, the role of emotional arousal has not been systematically investigated in neuroimaging studies. Moreover, the few behavioral studies that included examinations of arousal have produced inconsistent findings. For instance, a study investigating age-differences in memory for arousing and non-arousing words (Kensinger, 2008) showed that older adults remembered more positive than negative low-arousing words, whereas young adults showed the reverse pattern. There were no differences in the ratio of high-arousing positive vs. negative words remembered by young and older adults. A study examining age-differences in the relation between valence and arousal ratings (Keil and Freund, 2009) showed that in young adults both pleasantness and unpleasantness increased with emotional arousal, whereas in older adults low-arousing stimuli were experienced as most pleasant, and the high-arousing ones as most unpleasant. Finally, a study investigating age-differences in emotional reactions to stimuli differing in arousal and age-relevance (Streubel and Kunzmann, 2011) showed that older adults rated unpleasant low-arousing pictures as less unpleasant compared to young adults, while there were no age-related differences in unpleasant ratings for unpleasant pictures that were high in arousal. Despite these inconsistent findings, these behavioral studies indicate that age-related changes are more likely to influence the response to stimuli low in arousal. This idea is also suggested by recent neuroimaging evidence (St. Jacques et al., 2010) showing that older adults tend to rate some negative pictures as neutral more frequently than young adults (thus suggesting a negative-to-neutral shift in older adults). However, the latter study did not explicitly manipulate the level of arousal.

Previous evidence has shown that processing of low and high arousing emotional stimuli relies on distinct processes. On the one hand, low arousing negative stimuli activate more goal-driven processes, which tend to engage controlled, resource-demanding processes (Kensinger and Corkin, 2004) and the prefrontal cortex (PFC). On the other hand, high arousing information activates more stimulus-driven processes (Mather and Knight, 2006), which tend to capture attention automatically and require reduced cognitive effort (Dolan, 2002). There is evidence for the idea that the automatic processing of high-arousing stimuli is relatively preserved in older adults (Mather and Knight, 2006). One structure typically associated with the relatively automatic processing of emotional information is the amygdala. Amygdala, a region involved in basic emotion processing, is involved in two related and important abilities: (a) to rapidly detect emotional information presented with a rapid stream of stimuli (Anderson and Phelps, 1998) and (b) to detect emotional stimuli even when attentional resources are drained (reviewed by Dolan and Vuilleumier, 2003). These observations suggest that the role of the amygdala in emotional processing is not heavily dependent on the availability of extensive cognitive resources. Despite a relative structural preservation of the amygdala in healthy aging (Allen et al., 2005; Brabec et al., 2010), functional magnetic resonance imaging (fMRI) evidence reveals different patterns of activation in the amygdala. For instance, some fMRI studies have found an age-related decrease in activation in response to negative stimuli (Iidaka et al., 2002; Gunning-Dixon et al., 2003; Mather et al., 2004; Tessitore et al., 2005; Erk et al., 2008). One study reported robust amygdala activity in both young and older adults during the perception of novel fearful faces (vs. familiar neutral ones) (Wright et al., 2006), while another study found enhanced amygdala activity to negative compared to neutral stimuli in both young and older adults (St. Jacques et al., 2010). Importantly, the latter study also showed that the amygdala activation in older adults involved overlapping areas with young adults, thus suggesting that amygdala functions similarly in healthy young and older adults. This makes it unlikely that an age-related decline in the amygdala (as suggested by the aging-brain model: Cacioppo et al., 2011) would account for the reduced amygdala activity to negative stimuli in older adults reported in some of the previous studies.

A more probable explanation for the inconsistent findings regarding age-related differences in amygdala activation to negative stimuli may be that the pictures used in those studies differed in emotional arousal. This explanation is in line with previous evidence in older adults showing (a) preserved amygdala responses to positive (Mather et al., 2004) or novel stimuli (Moriguchi et al., 2011), (b) reduced responses selectively to negative stimuli, and (c) decreased amygdala activity for the trials in which older participants subjectively experienced negative pictures as neutral (negative-to-neutral shift), but not for the negative pictures subjectively rated as negative (St. Jacques et al., 2010). Taken together, these findings suggest the possibility of age-related differential engagement of the amygdala by stimuli with different levels of arousal, but again such manipulation has not been previously used in conjunction with brain imaging recordings.

Decreased amygdala activity, combined with increased activity in emotion control regions, such as the medial PFC and the adjacent anterior cingulate cortex (ACC), might also be the result of a greater focus on emotion regulation goals. According to the Socioemotional Selectivity Theory (SST; Scheibe and Carstensen, 2010), older adults tend to prioritize social and emotional well-being goals over other goals, potentially as a result of shrinking time horizons. The increased salience of emotional goals may enhance older adults' motivation to regulate the emotions experienced in everyday life, either by selecting stimuli and situations that minimize negative emotions (Blanchard-Fields et al., 2004) or by using emotion regulation strategies (Gross et al., 1997). The “cognitive-control model” extension of the SST (Mather and Knight, 2005; Kryla-Lighthall and Mather, 2009) posits that such negativity avoidance is the result of older adults' greater chronic focus on emotion regulation goals. Consistent with the cognitive control model, a number of studies reported increased activity in the medial PFC/ACC to negative compared to neutral stimuli in older compared to young adults (Gunning-Dixon et al., 2003; Williams et al., 2006; Leclerc and Kensinger, 2008; Roalf et al., 2011). Moreover, studies also showed that the increased activity in medial PFC/ACC was accompanied by decreased activity in the amygdala in older, but not in young adults, during the anticipation of monetary loss (Larkin et al., 2007) and during evaluation of negative stimuli (St. Jacques et al., 2010). Notably, functional connectivity between these regions was greater in older compared to young adults, thus suggesting that medial PFC and ACC might be involved more generally in subcortical emotion regulation.

This interpretation is consistent with studies showing interactions between similar medial PFC/ACC regions and the amygdala, when older adults voluntarily decreased emotional responses to negative stimuli (Urry et al., 2006; Winecoff et al., 2011). Given that medial PFC shows stronger activity when adults (a) are told to up-regulate positive emotion and down-regulate negative emotion (Ochsner et al., 2004) and (b) spontaneously regulate their emotions (e.g., Drabant et al., 2009), the increased medial PFC/ACC activity seen in older adults while processing emotional stimuli might reflect their spontaneous engagement of emotion regulation operations. Moreover, negative correlations between the ventral ACC and amygdala when evaluating negative pictures (according to IAPS standardized norms) as neutral (negative-to-neutral shift; St. Jacques et al., 2010) suggest that the spontaneous emotion regulation in older adults might be limited to low arousing stimuli.

The present event-related fMRI study used a broader range of negative emotional stimuli (low, medium, and high arousing) to test the hypothesis that emotional functioning in aging is modulated by the level of arousal, and to identify the associated neural correlates. The focus was on the ACC/vmPFC and the amygdala. Participants were presented with negative and neutral pictures, which they rated for emotional content, while fMRI data were recorded. Based on the above review, we made the following four predictions. First, from the evidence showing a negative-to-neutral shift in older adults' ratings, we predicted lower ratings to the low arousing negative pictures in older adults. Second, we predicted that common engagement of the amygdala in young and older adults would be specific to high arousing pictures, reflecting preserved responses to high arousing stimuli in older adults. Third, we predicted that opposing patterns of response in the ACC/vmPFC (increased) vs. amygdala (decreased) would be specific to low arousing stimuli. Finally, consistent with a role of the vACC/vmPFC in spontaneous emotional regulation, we explored the possibility that increased activity in this region would be linked to reduced ratings for low arousing negative stimuli in older adults.

## Methods

### Participants

The subject sample comprised 18 young adults between the ages of 18 and 32 years (10 females, mean age = 23.61, *SD* = 4.19) and 16 older adults between the ages of 59 and 84 years (11 females, mean age = 68.56, *SD* = 6.98). Participants were healthy, right-handed, native English speakers. As part of the initial screening, participants completed a number of questionnaires and cognitive measures. However, in the present study, these were used only for inclusion/exclusion purposes. Also as part of the initial screening, people with history of psychiatric and/or neurological conditions, uncontrolled hypertension, and subjects with history of alcohol or drug abuse were excluded from participation. It has been estimated that fMRI data from 18 subjects are needed to detect reasonable effect sizes with a power of 0.8 and an alpha of 0.05 (Huettel and McCarthy, 2001; Desmond and Glover, 2002). We collected data from more participants, but due to data attrition (e.g., ratings not recorded, response box problems, participants feeling uncomfortable in the scanner) only data from 18 young and 16 older participants are used for analyses. The age groups were matched on demographic variables, including education (see Table 1). All participants provided written informed consent under a protocol approved by the Institutional Ethics Review Board.

**Table 1:** **Summary of demographic characteristics for young and older groups**.

	**Younger**	**Older**	***p***
*n*	18	16	
Male/Female	8/10	5/11	
Age range	18–32	59–84	
Mean age (*SD*)	23.61 (4.19)	68.56 (6.98)	<0.001
Years of education (*SD*)	14.11 (2.22)	14.19 (2.97)	0.99

SD, standard deviation.

### Materials

Stimuli presented during the scanning session consisted of 180 pictures (90 negative and 90 neutral) selected from the IAPS (Lang et al., 1997). Additional neutral pictures were selected from other sources (Yamasaki et al., 2002), to equate the emotional pictures for visual complexity and human content. The negative picture set was further divided into equal numbers of low, medium, and high arousing negative pictures, based on their normative scores. Valence has not been held constant across the three levels of arousal in the negative pictures. Information about the pictures could be received from the corresponding author.

The focus on negative emotional pictures in the present study was justified by the following reasons. First, previous evidence shows that the influence of arousal on age differences is more pronounced for negative than for positive stimuli (Kensinger, 2008). Second, previous behavioral results show that positive stimuli are generally more difficult to interpret, due to the lack of consistency of subjective ratings for positive pictures with the normative ratings in the older participants (St. Jacques et al., 2010). Such inconsistencies are even more evident when considering arousal, as older adults tend to rate low-arousing pleasant pictures as more pleasant than high-arousing pleasant pictures (Streubel and Kunzmann, 2011). Thus, it would be difficult to meaningfully interpret the fMRI data based on the behavioral results. Third, to match the level of arousal in negative stimuli, the positive stimuli should have included a large number of pictures with radical sport and erotic content, which are processed differently in older adults (see Backs et al., 2005).

### Procedure

The pool of 180 pictures was divided into sets of 30 pictures (15 negative and 15 neutral pictures in each set), which were randomly assigned to six study blocks. The block orders were randomly assigned to the participants. To avoid mood induction, pictures were pseudo-randomized so that no more than three pictures of the same valence were consecutively presented. Functional MR images were recorded while participants viewed and rated negative and neutral images. Each picture was presented on the screen for 4 s, and then was removed to minimize confounding effects of eye movements associated with prolonged scanning of images. Participants were asked to watch the pictures and rate their subjective emotional experience triggered by the pictures on an 8-point scale (1 = neutral, 8 = extremely negative). The rating scale was presented at the bottom of each picture. The screen containing the picture and rating scale was followed by a fixation cross, presented on the screen for 12 s. Participants were instructed to rate the pictures only after they were aware of the content of the picture and of their emotional response to the picture. Participants were encouraged to try and rate the pictures while they were on the screen. However, participants were also told to respond during the fixation screen if they needed more time to rate the picture. Participants first completed a run in which they were instructed to experience any feelings or thoughts the pictures might trigger. The first run was intended to collect data on participants' spontaneous processing and evaluation of negative information with varying degrees of arousal. The following runs were intended to collect data on participants' responses following the induction of the goal to regulate emotion (Dolcos et al., 2011). Given the present focus on spontaneous processing of emotional information in young and older adults, the results reported here resulted from analyses performed on the data collected during the first run, before the induction of the goal to regulate emotions. The results of the emotion regulation manipulation are the focus of a different report.

### Scanning

fMRI data were recorded using a 1.5 Tesla Siemens Sonata scanner. The anatomical images were 3D MPRAGE anatomical series (repetition time, *TR* = 1600 ms; echo time *TE* = 3.82 ms; field of view, FOV = 256 × 256 mm; number of slices = 112; voxel size = 1 × 1 × 1 mm), and the functional images consisted of series of images acquired axially using an echoplanar sequence (*TR* = 2000 ms; *TE* = 40 ms; FOV = 256 × 256 mm; number of slices = 28; voxel size = 4 × 4 × 4 mm; flip angle = 90°; T2^*^-weighted images), thus allowing for full-brain coverage. Stimuli were projected on a screen directly behind the participant's head within the scanner, which participants viewed through a mirror. Responses were recorded using a 4-button response box placed under the participant's right hand; ratings 5–8 were indicated by the participants with double-clicks on buttons 1–4, respectively. Double-clicking to rate the higher-arousing negative pictures (to indicate a rating of 5–8) might lead to higher activation in motor areas, compared to the lower-arousing pictures, which required only one button click (ratings of 1–4). However, analyses looking at this issue showed no significant differences in the motor areas between the high and low arousing pictures in the two groups.

### fMRI data analysis

Standard pre-processing steps included quality assurance, TR alignment, motion correction, co-registration, normalization and smoothing (8 mm full-width half maximum isotropic kernel). Motion parameters calculated during the realignment were included as parameters of no interest to control for movement artifacts. For individual analyses, each event was modeled by the canonical hemodynamic response function (hrf) and its temporal derivative. The hemodynamic response can potentially show age-related differences (for review, see Dennis and Cabeza, 2008), but in the present study these differences were not problematic because we examined the effects of relative activity between task conditions (Buckner et al., 2000; Huettel et al., 2001; St. Jacques et al., 2010). The general linear model, as implemented in SPM2, was used to model the effects of interest and other confounding effects (e.g., magnetic field drift). Individual analyses produced whole-brain activation maps for the contrasts of interest. These individual contrasts were then entered into group-level random-effects analyses, which allowed investigation of the common and dissociating effects of negative and neutral pictures on brain activity engaged by young and older adults.

The main goal of the study was to investigate age-related differences in the neural correlates of evaluating emotional information with different levels of arousal. The focus was on the role of regions involved in basic emotion processing (amygdala) and emotion control (ACC/vmPFC). To accomplish this goal, analyses were performed to identify the common set of brain regions engaged by both young and older adults, through conjunction analyses. To examine our a priori hypothesis regarding the amygdala, we used anatomical ROI masks derived from the Wake Forest University Pick Atlas toolbox (Dolcos et al., 2004). For the amygdala, an intensity threshold of *p* < 0.05 uncorrected and an extent threshold of five contiguous voxels were used. The conjunction map for the amygdala was defined as [(Negative_Old_ > Neutral_Old_) ∩ (Negative_Young_ > Neutral_Young_)], and calculated using the ImCalc feature in SPM. Thus, the conjoint probability of the conjunction map for the amygdala was 0.0025 (Fisher, 1950). In this context, we would like to emphasize the advantages of using the conjunction procedures. The conjunction procedure was performed using the Minimum Statistic compared to the Conjunction Null (MS/CN; Nichols et al., 2005), where a voxel/cluster only survives if it is significant in each independent map included in the conjunction analysis. While corrections for multiple comparisons, such as false discovery rate (FDR) and family-wise error rate (FWER), offer conservative approaches to controlling for Type I errors, they are (especially FWER) prone to Type II errors (Lieberman and Cunningham, 2009). Therefore, when used in conjunction, the statistical and extent thresholds used for the current analyses offer the best balance between Type I and II errors and result in a statistical value that is within the acceptable criterion for publication (Forman et al., 1995; Lieberman and Cunningham, 2009).

Further analyses were performed to identify dissociable sets of brain regions showing greater sensitivity to negative than to neutral pictures across groups, using ANOVAs and two-sample *t*-tests. Analyses were also performed separately for the low, medium and high arousing negative stimuli. Finally, to further elucidate the role of the brain regions showing age-related differences in response to negative and neutral stimuli, brain-behavior relations were investigated by examining co-variations of their neural responses with behavioral ratings. Behavioral measures were correlated with mean statistics after identifying clusters of activation at the group level. For all analyses, an intensity threshold of *p* < 0.005 uncorrected and an extent threshold of 10 contiguous voxels were used, except for the amygdala where an intensity threshold of *p* < 0.05 and an extent threshold of five contiguous voxels were used. An intensity threshold of *p* < 0.005 uncorrected coupled with an extent threshold of 10 voxels is typically considered a good trade-off between Type I and Type II errors (Lieberman and Cunningham, 2009). We also corrected for multiple comparisons in two ways. We applied two levels of FDR corrections: one corresponding to a *p*-value of 0.05 for each anatomical ROI (see **Table 3**), and the other corresponding to a *p*-value of 0.05 for each functional cluster.

## Results

### Behavioral results

#### Reduced ratings to low arousing negative pictures in older adults

First, a Valence (Negative and Neutral) × Age Group (Young and Older) repeated-measures ANOVA with Valence as a within-subject factor and Age Group as a between-subjects factor revealed a main effect of Valence [*F*_(1, 32)_ = 247.38, *p* < 0.001]. The Valence × Age Group [*F*_(1, 32)_ = 1.66, *p* = 0.21] interaction was not significant. Second, separate analyses showed the expected effect of valence, with the negative pictures being rated as more negative than the neutral pictures, both by the young (*t* = 17.28, *p* < 0.001) and the older (*t* = 7.97, *p* < 0.001) adults. Third, an Arousal (Negative Low, Medium, and High) × Age Group (Young and Older) repeated-measures ANOVA with Arousal as a within-subject factor and Age Group as a between-subjects factor revealed a main effect of Arousal [*F*_(1, 32)_ = 46.86, *p* < 0.001]. The Arousal × Age Group [*F*_(1, 32)_ = 0.77, *p* = 0.47] interaction was not significant. Fourth, planned comparisons revealed a trend in the age-related comparison of low-arousing negative pictures, consistent with our first prediction that the ratings for low-arousing negative pictures will be lower in the older (*M* = 3.88, *SD* = 1.19) than in the young (*M* = 4.62, *SD* = 1.29) adults (*t* = 1.72, *p* = 0.096; one-tailed *p* = 0.048). There were no age-related differences in the ratings for the high and medium arousing negative pictures, nor in the ratings for the neutral pictures (see Table 2). Analyses also showed no age-related differences in the ratings for Negative High vs. Negative Low, and Negative Medium vs. Negative Low.

**Table 2:** **Behavioral ratings for young and older groups**.

	**Age group**		***t***	***p***
	**Young (*n* = 18**)	**Older (*n* = 16**)		
NegHi	6.55 (1.08)	5.68 (1.82)	1.68	0.106
NegMed	5.58 (1.38)	5.18 (1.59)	0.80	0.425
NegLo	4.62 (1.29)	3.89 (1.19)	1.72	0.096
*NegAll*	*5.59 (1.11)*	*4.91 (1.39)*	1.56	0.128
*NeuAll*	*1.88 (0.55)*	*1.77 (0.60)*	0.55	0.584

Values before parentheses indicate the means. Values in parentheses indicate standard deviations. Reported p-values are two-tailed.

### fMRI results

#### Common engagement of the amygdala in young and older groups driven by high arousing stimuli

**Figure 1:**
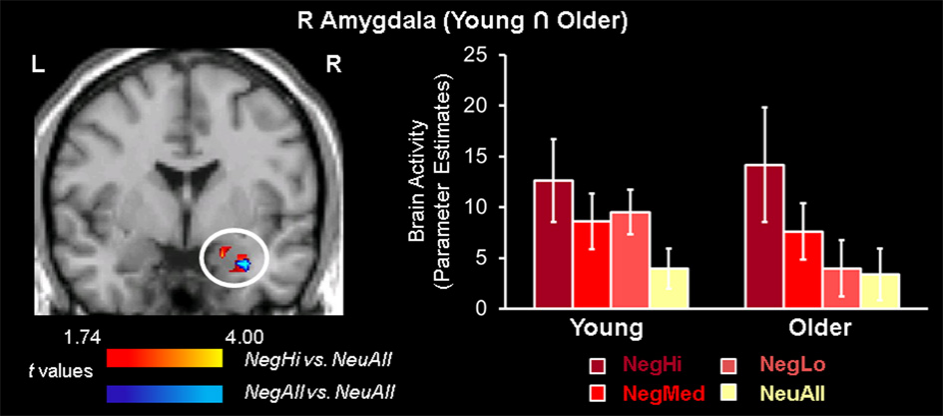
**Common amygdala activation for high arousing stimuli in young and older adults**. Common engagement of the right amygdala by young and older groups was identified using conjunction analysis based on a region of interest approach, where both young and older groups showed common activity for negative vs. neutral (blue area) and high arousing negative vs. neutral (red area) stimuli (*p* = 0.0025). The bar graph illustrates the parameter estimates corresponding to the peak voxel in the area of R AMY commonly activated by young and older adults for NegAll vs. NeuAll contrast. L, left; R, right; NegHi, Negative High Arousal; NegMed, Negative Medium Arousal; NegLo, Negative Low Arousal; NegAll, Negative All; NeuAll, Neutral All.

Conjunction analyses of brain activity associated with the evaluation of negative and neutral pictures identified an area in the right amygdala that was commonly engaged by both age groups (see Table 3; Figure 1). Consistent with previous evidence showing common engagement of the amygdala to negative compared to neutral stimuli by both age-groups (St. Jacques et al., 2010), we did not find significant differences in this region in an ANOVA examining Valence (Negative and Neutral) × Age Group (Young and Older) interaction. Analyses looking at the effect of arousal revealed that the common engagement of the right amygdala in the two age-groups was driven by the high arousing negative stimuli, which also activated left amygdala in both groups. Further analyses identified amygdala activation to low, medium, and high arousing negative stimuli in younger adults, but only to high and medium arousing negative stimuli in older adults. However, there were no overlapping areas in the brain regions engaged by the low and medium arousing pictures. The Arousal (NegLo vs. NeuAll, NegMed vs. NeuAll, and NegHi vs. NeuAll) × Age Group (Young and Older) interaction was not significant in the amygdala region commonly engaged by young and older adults in response to negative compared to neutral pictures, but it was significant (*F* = 4.55, *p* < 0.05) for the peak voxel showing decreased amygdala activity to low-arousing stimuli in the older compared to young adults (discussed below). Although the interaction was not significant in the amygdala region commonly engaged by young and older adults in response to negative compared to neutral pictures, our findings are still consistent with the idea that the overlap is more likely to occur in high-arousing stimuli, and with our prediction that amygdala activity will be associated with minimal age-related differences for high arousing negative stimuli, but will show age-related differences for the low-arousing negative stimuli. This differential impact is also reflected in the results of the follow-up pairwise comparisons showing age-related differences in the amygdala to low, but not to medium and high arousing negative pictures. *T*-tests comparing amygdala activation to high arousing negative stimuli revealed reduced activity to the high arousing negative pictures in temporal, occipital, parietal, and cingulate areas, and to the medium arousing pictures in occipital areas (see Table 4) in older compared to the young adults. In addition to the right amygdala, high arousing stimuli also engaged a common area in the extra striate cortex (Talairach coordinates: *x* = 48, *y* = −74, *z* = 7), in both young and older adults. As described below, low arousing pictures were associated with different patterns of activity in young and older adults.

**Table 3:** **Common amygdala activity for negative vs. neutral stimuli**.

**Brain region**	***H***	**Voxels**	**Talairach coordinates**			***t***
			***x***	***y***	***z***	
**YOUNG ∩ OLDER (NegAll vs. NeuAll)**						
Amygdala	R	6	28	−1	−20	**3.86**
**YOUNG ∩ OLDER (NegHi vs. NeuAll)**						
Amygdala	L	6	−20	−5	−17	**3.98**
Amygdala	R	22	16	−5	−17	3.11^*^
			28	−1	−20	2.86^*^

H, Hemisphere. t-values represent the multiplied score from the conjunction analysis of young and older groups. Bolded t-values indicate the voxels surviving a voxel-level threshold of p < 0.05 (FDR-corrected).

* Denotes significant difference at a cluster-level threshold of p < 0.05 (FDR-corrected).

**Table 4:** **Dissociable brain activity for negative vs. neutral stimuli by arousal levels in young and older adults**.

**Contrast**	**Brain region**	**BA**	***H***	**Voxels**	**Talairach coordinates**			***t***
					***x***	***y***	***z***	
**YOUNG > OLDER**								
**High arousal (NegHi vs. NeuAll)**								
Cuneus		18	R	26	12	−85	19	4.26
					8	−92	19	3.71
Precuneus		19	R	12	28	−60	36	3.94
Middle temporal gyrus		19	R	21	48	−61	14	3.85
Cingulate gyrus		32	L	17	−4	17	32	3.69
Postcentral gyrus		2	R	10	51	−21	42	3.66
Middle temporal gyrus		19	L	18	−44	−61	14	3.60
Middle temporal gyrus		37			−51	−62	3	2.83
**Medium arousal (NegMed vs. NeuAll)**								
Middle occipital gyrus		18	L	21	−32	−89	4	3.47
					−24	−89	15	3.38
**Low arousal (NegLo vs. NeuAll)**								
Amygdala		34	L	11	−20	−1	−10	2.49^*^
					−32	−5	−17	2.10^*^
Amygdala		28	R	18	16	−4	−10	2.44^*^
		34			28	3	−14	1.96^*^
Inferior frontal gyrus		47	R	34	44	11	−4	4.81
Superior temporal gyrus		41	R	13	40	−35	9	4.02
					48	−31	5	3.17
					40	−38	16	2.82
Middle temporal gyrus		39	R	23	48	−61	21	3.85
					44	−54	14	3.31
					48	−62	10	2.99
Superior frontal gyrus		9	R	26	40	40	31	3.79
					16	52	38	3.78
					4	56	38	3.67
Superior frontal gyrus		9	L	22	−28	52	34	3.77
					−12	52	38	3.62
Middle occipital gyrus		19	L	14	−28	−77	11	3.75
					−32	−85	8	3.43
					−24	−89	15	3.09
Inferior frontal gyrus		47	L	20	−48	42	−9	3.75
					−51	30	−5	2.94
					−55	35	2	2.86
Middle frontal gyrus		8	R	32	36	22	47	3.68
					48	21	39	3.67
					32	6	58	3.25
Superior temporal gyrus		38	L	40	−44	11	−14	3.64
					−40	23	−11	3.47
					−51	7	−7	3.32
**OLDER > YOUNG**								
**Low arousal (NegLo vs. NeuAll)**								
Anterior cingulate gyrus		32	L	39	−16	36	13	**4.22**
					−12	43	−2	2.42
		32	R	8	8	40	16	1.88

BA, Brodmann's area; H, Hemisphere. A threshold of p < 0.005 (uncorrected) was used. Bolded t-values indicate the voxels surviving a cluster-level threshold of p < 0.05 (FDR-corrected).

* Denotes significant difference at p < 0.05 (uncorrected).

#### Opposing spontaneous responses in rostral/ventral ACC and amygdala activity in older adults driven by low-arousing pictures

**Figure 2:**
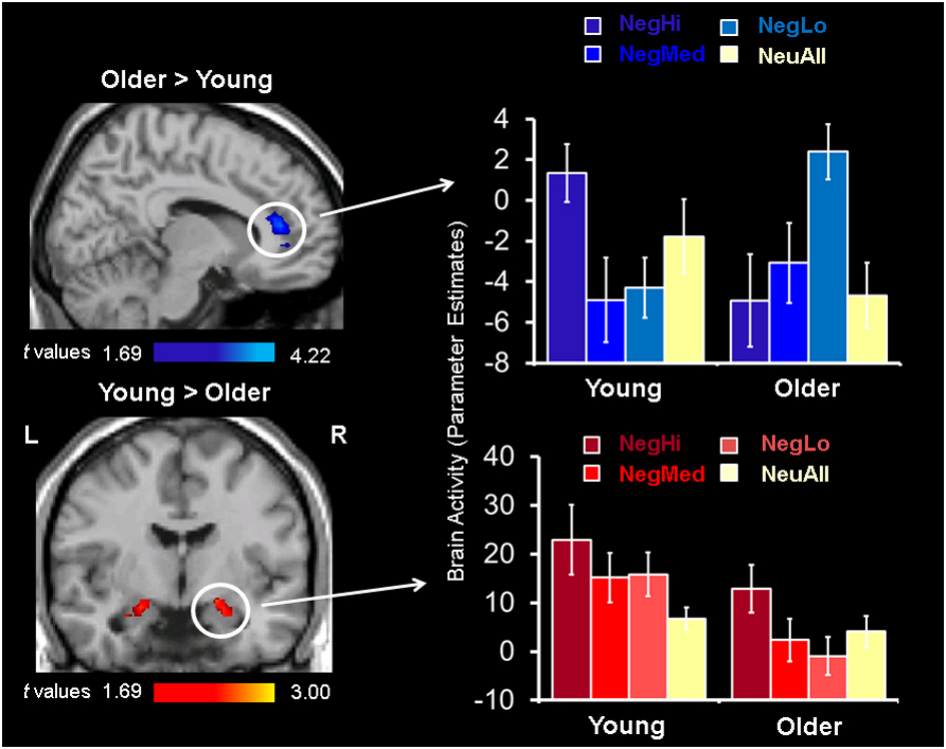
**Opposing patterns of response in rostral/ventral ACC and AMY for low arousing stimuli**. Increased activity in rostral/ventral ACC (top sagittal image), and decreased activity in the amygdala (bottom coronal image) in older compared to young adults was identified using two-sample *t*-tests between activation maps for NegLo vs. NeuAll contrast in the two groups. The bar graphs on the right illustrate the parameter estimates corresponding to the peak voxels in the area of ACC and AMY showing increased activity to low arousing negative stimuli in the young and older groups. L, left; R, right; NegHi, Negative Hi Arousal; NegMed, Negative Medium Arousal; NegLo, Negative Low Arousal; NeuAll, Neutral All.

Confirming our third prediction, targeted two-sample *t*-tests revealed an age-related increase to low arousing stimuli in the rostral/ventral ACC (Tables 4, 5). In older adults this region showed activation, whereas in young adults it showed deactivation. Deactivation in this area is not surprising, as a variety of previous fMRI studies have shown that deactivation in medial frontal areas is rather common during tasks that entail goal-directed behavior (Frankenstein et al., 2003). Deactivations in this region typically occur when resources are allocated to other brain regions, possibly to facilitate task-relevant processing (Frankenstein et al., 2003). Importantly, this region partially overlapped with the ACC region showing a significant Arousal × Age Group interaction (*F* = 12.70, *p* < 0.005). Targeted two-sample *t*-tests also revealed an age-related decrease in the bilateral amygdala activity, for the low arousing pictures (see Figure 2). Moreover, the peak voxel showing decreased amygdala activity to low arousing stimuli in older compared to the young adults also survived the Arousal × Age Group interaction (*F* = 4.55, *p* < 0.05). As illustrated in Figure 2, rostral/ventral ACC activation was significant only for the older group, whereas the amygdala activation was significant only for the young group. In addition, young adults showed increased activity in frontal, temporal, and occipital areas (see Table 4).

**Table 5:** **Mean parameter estimates for peak voxel activity in anterior cingulate cortex (ACC) and amygdala (AMY) for young and older groups**.

**Brain region**		**Age group**	
		**Young (***n*** = **18**)**	**Older (***n*** = **16**)**
ACC	NegHi	1.35 (1.42)	−4.93 (2.27)
	NegMed	−4.91 (2.08)	−3.08 (1.98)
	NegLo	−4.29 (1.48)	2.39 (1.35)
	NeuAll	−1.78 (1.83)	−4.68 (1.60)
AMY	NegHi	22.93 (7.18)	12.88 (4.92)
	NegMed	15.18 (5.07)	2.34 (4.34)
	NegLo	15.83 (4.44)	−0.93 (3.96)
	NeuAll	6.74 (2.29)	4.12 (3.20)

Values before parentheses indicate the means. Values in parentheses indicate the standard errors of the means. The mean parameter estimates were extracted in each of the four conditions against the baseline activity for each age group at the coordinates corresponding to the peak voxels for each of the ACC and AMY regions showing overlapping activity between (1) the Arousal × Age Group interaction and (2) the two-sample t-test targeting low arousing stimuli.

#### Age-related increased activity in ventral ACC/ventromedial PFC was linked to lower ratings for low arousing negative stimuli

**Figure 3:**
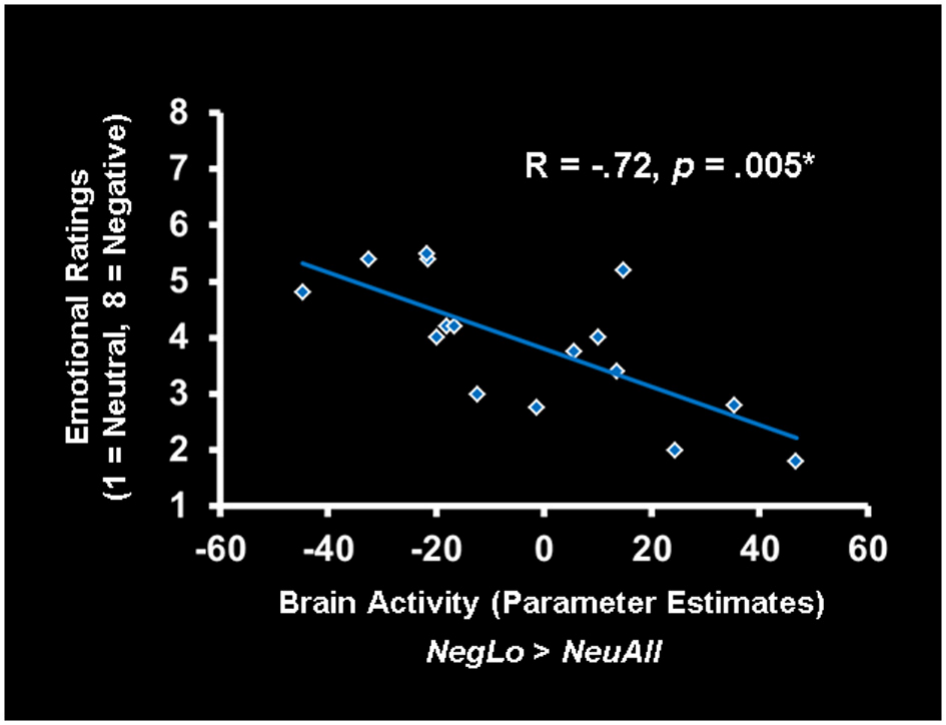
**Activity in the ACC was linked to reduced ratings for low-arousing stimuli in older adults**. In older adults, increased activity in the ventral anterior cingulate cortex (vACC)/ventro-medial prefrontal cortex (vmPFC) correlated negatively with the ratings for low arousing negative stimuli. The scatterplot is based on the parameter estimates corresponding to the peak voxel in the area of vACC/vmPFC showing negative correlation with the behavioral ratings. NegLo, Negative Low Arousal; NeuAll, Neutral All.

Co-variations of activity in the vACC/vmPFC with the behavioral ratings further elucidated the role played by this region in the evaluation of low arousing stimuli in older adults (see Figure 3). As illustrated in the figure, the vACC/vmPFC (Talairach coordinates: *x* = 0, *y* = 38, *z* = −9) activity was negatively correlated with the ratings for low arousing negative pictures in the older adults (*r* = −0.72, *p* < 0.005). That is, older participants who engaged this region when processing low arousing negative pictures also rated those pictures as less negative. Of note, portions of the vACC/vmPFC showing the negative co-variation with ratings for low arousing pictures in older adults partially overlapped with the vACC/vmPFC area showing stronger response to low arousing negative stimuli in older, compared to the young adults. The correlation between vACC/vmPFC and ratings was specific to ratings for low arousing negative pictures in older adults, as indicated by the significant difference between this correlation and the similar correlation for the young group (using the *r* to *z* transformation, *z* = −4.06, *p* < 0.0001). There were no other significant correlations between the vACC/vmPFC and ratings. The significant co-variations between other brain areas and behavioral ratings are presented in Table 6.

**Table 6:** **Co-variations between brain activity and the ratings of negative pictures by young and older adults**.

**(Contrast) × Ratings**	***r* value**	**Brain region**	**BA**	***H***	**Voxels**	**Talairach coordinates**			***t***
						***x***	***y***	***z***	
**YOUNG**									
(NegHi vs. NeuAll) × NegHi	−0.668	Inferior frontal gyrus	47	L	10	−20	31	−5	3.59
(NegLo vs. NeuAll) × NegLo	−0.726	Parahippocampal gyrus	35	L	14	−20	−35	−5	4.22
**OLDER**									
(NegHi vs. NeuAll) × NegHi	+0.803	Precuneus	7	L	56	−4	−53	43	5.04
	+0.770					0	−45	28	4.52
	+0.705		7	R		12	−52	43	3.72
	+0.778	Precentral gyrus	9	L	12	−36	21	39	4.64
	+0.769	Superior parietal lobule	7	R	16	28	−68	44	4.50
	+0.746	Precuneus	7	L	10	−12	−59	55	4.19
	+0.722					−12	−68	51	3.90
	+0.707	Medial frontal gyrus	8		12	0	45	38	3.74
	+0.707					0	56	30	3.74
	+0.669	Medial frontal gyrus	9	L		−4	40	31	3.37
	+0.690	Superior frontal gyrus	9	L	14	−8	60	26	3.57
	+0.679			R		8	63	15	3.45
(NegMed vs. NeuAll) × NegMed	−0.819	Inferior parietal lobule	40	L	11	−40	−29	42	5.34
	−0.686					−28	−37	42	3.52
	−0.797	Precentral gyrus	6	R	10	32	−9	56	4.94
	−0.783	Precentral gyrus	6	L	26	−24	−10	52	4.70
	−0.699					−16	−1	55	3.65
	−0.699					−24	−1	48	3.65
	−0.777	Precentral gyrus	6	L	13	−55	−2	41	4.62
	−0.774	Precuneus	7	R	26	8	−59	62	4.58
	−0.668					4	−56	51	3.35
	−0.751	Precuneus	7	L	11	−28	−71	55	4.25
	−0.749	Caudate nucleus		R	20	4	8	0	4.23
	−0.711			L		−4	4	−4	3.79
	−0.739	Lentiform nucleus		R	19	20	−4	8	4.11
	−0.712	Inferior parietal lobule	40	L	21	−44	−36	57	3.79
	−0.700					−40	−47	61	2.67
	−0.710	Superior temporal gyrus	22	R	10	60	−7	8	3.77
	−0.708					51	4	7	3.75
	−0.675					48	5	15	3.43
(NegLo vs. NeuAll) × NegLo	+0.750	Insula	13	R	12	28	−26	23	4.24
	+0.743					36	−45	28	4.22
	+0.767					32	−34	24	4.04
	+0.711	Supramarginal gyrus	40	L	11	−44	−45	32	4.16
	+0.653	Superior frontal gyrus	8	R	10	4	37	46	3.53
(NegLo vs. NeuAll) × NegLo	−0.577	Anterior cingulate	32	R	7	0	38	−9	4.11

BA, Brodmann's area; H, Hemisphere. A threshold of p < 0.005 (uncorrected) was used.

## Discussion

Despite substantial evidence supporting the idea of enhanced affective well-being and emotional stability in healthy aging, relatively little is known about the role of emotional arousal in this effect and the underlying brain mechanisms. The current study addressees this gap in the emotional aging literature by investigating the effect of emotional arousal on behavioral and neural responses in young and older adults. There were three main novel findings regarding the neural correlates. First, we showed that the common engagement of the right amygdala in young and older adults was driven by high arousing stimuli. Second, we showed that the opposing spontaneous pattern of increased activity in the rostral/ventral ACC and decreased activity in the right amygdala is specific to low arousing stimuli. Third, we linked the increased spontaneous activity in the vACC/vmPFC to older adults' reduced ratings for low arousing stimuli. These findings are discussed below.

### Common engagement of the amygdala in young and older groups driven by high arousing stimuli

The present study revealed that right amygdala activation in older adults had overlapping areas with that from younger adults, thus showing that both groups involve the same amygdala regions to process negative stimuli. Moreover, the present study advances previous findings (St. Jacques et al., 2010) by showing that the common engagement of the right amygdala is driven by high arousing stimuli. Decreased amygdala activity to low arousing stimuli in older adults might be attributed to a potential deterioration of this brain structure with age. However, our finding showing similar amygdala engagement by high arousing stimuli in both age groups provides evidence against such a hypothesis and confirms previous evidence showing that amygdala activity is preserved with age. Increased amygdala activation by high arousing negative emotional stimuli is consistent with previous behavioral findings suggesting that emotion processing is not impaired in aging (Mather and Knight, 2006), and with brain imaging evidence showing that aging is associated with robust functional activation of the amygdala (Wright et al., 2006; St. Jacques et al., 2010). Increased amygdala activation in response to high arousing stimuli is also consistent with previous behavioral evidence (Kensinger, 2008) showing minimal age-differences for high arousing stimuli (but identifying age-related differences for low arousing stimuli).

The present results are also in line with more recent theories of emotional aging (cognitive control model: Mather and Carstensen, 2005), which suggest that the relatively preserved emotional well-being in older adults is the result of age-related differences in the controlled processing of emotional information, and that processing of high arousing stimuli is less dependent on controlled processing compared to low arousing stimuli (Kensinger, 2008). Therefore, if the emotional well-being is caused by age-related declines in the controlled processing of emotional information, evaluation of low arousing stimuli, which relies more on controlled processes, would be more affected than the evaluation of the high arousing stimuli, which relies more on automatic processes. Given that (a) amygdala is involved in processing stimulus relevance for the goals and motivations of the perceiver (Cunningham and Brosch, 2012) and (b) right amygdala is involved in the initial, rapid, possibly automatic detection of an emotional stimulus (Gläscher and Adolphs, 2003), then (c) our results showing bilateral amygdala engagement in the evaluation of high arousing stimuli by both age groups suggest that this type of stimuli are automatically detected by both groups because of their increased personal relevance. The present findings showing dissociable responses in the commonly activated amygdala based on the level of arousal suggest that the age-related differences identified in previous studies may not be caused by an overall decline in the amygdala functioning, but rather by age-related changes in the intensity of the emotional stimuli to which amygdala is more receptive.

### Opposing spontaneous responses in rostral/ventral ACC and amygdala activity in older adults specific to low-arousing pictures

The present study also advances previous findings by showing that the age-related differences in the engagement of amygdala and rostral/ventral ACC are specific to low arousing negative stimuli. Older adults' reduced amygdala activation to low arousing negative pictures can be the consequence of many factors, including an age-related atrophy in neural systems important for processing negative stimuli, age-related decreases in psychophysiological responses to arousing stimuli (Tsai et al., 2000), or an age-related decline in the amygdala, which selectively diminishes emotional arousal in response to negative stimuli and decreases the experienced negative affect (the aging brain model; Cacioppo et al., 2011). However, in the present study, the decreased bilateral amygdala activity was accompanied by increased activity in the rostral/ventral ACC in older compared to the young adults. This opposing pattern of brain activity is consistent with previous findings showing decreased amygdala activity coupled with increased activity in cortical control regions during the perception and evaluation of negative stimuli (anterior ventral insula; Fischer et al., 2005; ventral and medial PFC; Tessitore et al., 2005), and with greater functional connectivity between the right amygdala and ACC in older compared to young adults (St. Jacques et al., 2010). Moreover, a similar region has shown a negative correlation with right amygdala on trials in which older adults subjectively experienced negative pictures (according to the IAPS standardized norms) as neutral, but not for negative pictures subjectively rated as negative (St. Jacques et al., 2010). This suggests a role of this region in reducing amygdala activity when regulation is successful. This evidence, together with our findings, indicate that older adults' reduced amygdala activation to low arousing pictures may be the result of their successful emotion regulation of this type of stimuli.

Taken together, our findings show enhanced activity in an emotional control region, the rostral/ventral ACC, coupled with decreased bilateral amygdala activity to low arousing negative stimuli, in a task in which participants were not explicitly instructed to down-regulate their emotional responses. This may reflect older adults' spontaneous engagement of emotion control regions to down-regulate low arousing negative emotions. Moreover, the negative association between vACC/vmPFC and behavioral ratings for low arousing stimuli, discussed below, reflects their success in regulating low arousing negative emotions. These results support the idea that older adults may have emotion regulation chronically activated (Mather and Carstensen, 2005; Nashiro et al., 2012), and that this effect is specific to low arousing negative emotions.

### Age-related increased activity in ventral ACC/ventromedial PFC linked to lower ratings for low arousing stimuli

Ventromedial PFC and the ACC play an important role in processing emotional information, and are particularly involved in the automatic regulation of emotional responses (for reviews see Bush et al., 2000; Phillips et al., 2003) during incidental emotion regulation paradigms, such as affect labeling and self-distraction from a fear-conditioning stimulus (Lieberman et al., 2007). Unlike other frontal regions, which thin dramatically with aging (Fjell et al., 2009), vmPFC/ACC maintain their cortical thickness in normal aging, which suggests that some of the neural circuitry critical for emotion regulation is well preserved in older adults. In the present study, evaluation of low arousing negative stimuli was associated with increased vACC/vmPFC activity in older compared to the young adults. Moreover, activity in these areas was negatively associated with the behavioral ratings for low arousing stimuli in older adults, further supporting a role of this region in effective spontaneous regulation of this type of emotions.

Although our study contributes important novel information, it also has some limitations. One limitation concerns the size of our subject sample in the older group, which although allowed identification of robust findings, was slightly smaller than the optimal fMRI sample size of 18 suggested for investigations of brain-behavior relations (Lieberman et al., 2009). However, the size of our samples was comparable to that involved in other investigations of age-related differences (e.g., St. Jacques et al., 2010). Although we took measures to minimize multiple comparisons, we recognize the importance of replicating the findings in a larger sample. There were also different age-spans in the young (14 years) and the older (25 years) groups, which potentially could have led to greater variance in the older adults group. Finally, involving only negative stimuli and manipulating only the level of arousal limits the generalizability of our results to negative stimuli with different degrees of arousal. Future studies should investigate whether the effects identified in the present study are confirmed for positive stimuli and in investigations that manipulate independently emotional valence and arousal.

## Conclusion

In the present study, we examined the effect of arousal as a potential factor influencing the enhanced affective well-being and emotional stability commonly associated with healthy aging. There were three novel findings regarding the neural correlates of emotion processing: (a) right amygdala was commonly engaged by young and older adults only during the evaluation of high arousing stimuli, (b) amygdala and rostral/ventral ACC showed an opposing pattern of activity for low arousing stimuli in older compared to young adults, and (c) the engagement of the ventral ACC/vmPFC in older adults reflected successful regulation of low arousing negative stimuli. These findings highlight the important effect of arousal on age-related emotional processing, suggesting that aging is associated with preserved emotional processing of high arousing negative information, and with altered processing of low arousing negative information. By showing that older adults engage more automatic processes when evaluating high arousing negative information, and more controlled, resource-demanding processes in response to low arousing negative information, the present study advances our understanding of the neural correlates underlying the enhanced emotional well-being in healthy aging. Moreover, by linking the spontaneous engagement of the emotion control regions in older adults to reduced subjective experiencing of low arousing negative information, the present study provides further evidence supporting the idea that emotion regulation is chronically activated in healthy aging, and clarifies that this effect is specific to low arousing negative information. This new evidence highlights the need of adopting a comprehensive approach that takes into consideration both the valence and arousal in examining emotional aging.

### Conflict of interest statement

The authors declare that the research was conducted in the absence of any commercial or financial relationships that could be construed as a potential conflict of interest.
